# Intrafamilial Variability of Early-Onset Diabetes due to an *INS* Mutation

**DOI:** 10.1155/2011/258978

**Published:** 2011-06-30

**Authors:** Siri Fredheim, Jannet Svensson, Sven Pørksen, Lars Hansen, Torben Hansen, Oluf Borbye Pedersen, Henrik Bindesbøl Mortensen, Fabrizio Barbetti, Lotte Brøndum Nielsen

**Affiliations:** ^1^Department of Paediatrics, Herlev University Hospital, Ndr Ringvej 75, 2730 Herlev, Denmark; ^2^The Faculty of Health Sciences, University of Copenhagen, Denmark; ^3^Steno Diabetes Center, Niels Steensens Vej 2, 2820 Gentofte, Denmark; ^4^Laboratory of Monogenic Diabetes, Bambino Gesù Children's Hospital, Tor Vergata University Hospital, Viale Oxford, 81 00133 Rome, Italy

## Abstract

*Aim*. The objective of this study was to describe the clinical characteristics of two siblings and their father carrying a C95Y mutation in the insulin (*INS*) gene. *Methods/Results*. A Danish patient, his sister, and his father were identified to carry the C95Y mutation in the preproinsulin molecule causing permanent neonatal diabetes. All three were diagnosed before 29 weeks of age, were born at term with near-normal birth weight, and were negative for GAD, ICA, IA-2, and IAA autoantibodies. The daily insulin requirement the first six months after diagnosis was <0.5 U kg^−1^ day^−1^ for both children. The father, insulin treated for over 40 years, has bilateral preproliferative retinopathy. *Conclusions*. These three cases further confirm the essential features of diabetes caused by *INS* mutations with proteotoxic effect. We conclude that patients with similar features must be investigated for mutations of *INS* gene.

## 1. Introduction

Neonatal diabetes (ND) is a rare monogenic disorder that can be clinically divided in two forms, transient neonatal diabetes mellitus (TNDM) and permanent neonatal diabetes mellitus (PNDM), caused by defects in several genes. Recent studies have estimated the incidence of PNDM at 1 in about 200,000–250,000 live births [1–3]. The most common causes of PNDM are mutations in the genes encoding for the two subunits of ATP-sensitive potassium channel (K_ATP_) of the pancreatic beta cell, *KCNJ11* and *ABCC8*, accounting for about 50 and 10% of all cases, respectively. However, heterozygous, dominant mutations in the insulin gene (*INS*) are also frequently found in patients with PNDM, representing about 15–20% of all cases [4–7].

Though heterozygous, gain-of-function (toxic) mutations of the *INS* gene are mainly detected in patients with infancy-onset diabetes (i.e., within six months from birth), patients identified with these mutations may present with a wide heterogeneity of diabetes presentation with age of onset varying from 0 to 20 years of age [4–8]. The first report was published by Støy et al. describing 10 heterozygous mutations in the *INS* gene in sixteen probands with neonatal diabetes [6]. Most of the probands were diagnosed before the age of six months, but few of them were diagnosed between six months to one year of age; in addition, one father was diagnosed at the age of 30 years with mild type 2 diabetes. This study has been followed by other papers confirming heterogeneity of diabetes presentation with age of onset varying from 0 to 20 years of age [4, 5, 7, 8]. Colombo et al. compared clinical findings of patients with PNDM due to *INS* or *KCNJ11* mutations and found that detectable or high C-peptide level, near-normal birth weights, and older age at onset of disease were recurring features of patients with *INS* mutations as compared to patients carrying a *KCNJ11* mutation [7]. 

Here we report the clinical characteristics of a family with a C95Y mutation in the *INS* gene and the assessment of late onset diabetes complications in the adult family member.

## 2. Patients and Methods

### 2.1. Subjects

We studied the son and the daughter of a white Caucasian male originally from New Zealand, married to a Danish woman, whose insulin gene mutation has previously been described by Colombo et al. [7].

### 2.2. Methods

*INS* gene sequencing was performed by the laboratory at Steno Diabetes Center, Gentofte, Denmark following the method described by Stoy et al. [6].

The residual beta-cell function (assessed by stimulated C-peptide) was measured 4 weeks after onset (and 52 weeks in the patient) by a Boost test (6 mL/kg of Boost/Sustacal (Mead Johnson, Evansville, IN, USA)). The test was performed in the morning after at least 8 hour fasting. In agreement with the DCCT protocol, capillary glucose was measured at time 0 and venous C-peptide and glucose at 90 min after ingestion of Boost.

## 3. Results

The patient, a 28-week-old boy, first child born to non-consanguineous parents, was referred to us at Glostrup University Hospital, for polydipsia and polyuria. Family history included the father who was diagnosed with PNDM and patient's grand-grandfather with type 2 diabetes. The antenatal period was uncomplicated. He was born at term, by normal vaginal delivery (Table 1). The physical examination was unremarkable. Glycated haemoglobin (HbA1c) upon admission was 13.5% (reference range 4.3–5.8). He tested negative to all T1D-related autoantibodies, that is, islet cell autoantibodies (ICA), glutamic acid decarboxylase 65 antibodies (GAD 65), insulinoma antigen-2 antibodies (IA-2A), and insulin autoantibodies (IAA). DNA sequencing identified the same *INS*/C95Y mutation previously found in his father [7]. He was initially treated with a subcutaneous insulin dose <0.5 U kg^−1^ day^−1^ (twice daily) until five months after disease onset where we observed fluctuations in HbA1c (Table 1). Thus, he was first switched to multiple daily injections (MDIs) at two years of age and then to insulin pump therapy at the age of three. 

His younger sister was diagnosed at 10 weeks. She was born at term after a normal pregnancy by vaginal delivery (Table 1). Blood glucose measured at home by her mother was 15 mmol/L. The physical examination at admission was unremarkable. HbA1c was determined to be 5.4% of total haemoglobin which was considered falsely normal due to the presence of foetal haemoglobin. She was negative for all T1D-related autoantibodies, and we confirmed that she inherited *INS*/C95Y mutation from her father. She was started on subcutaneous insulin twice daily with a subsequent immediate improvement in blood glucose. Like her brother, her insulin requirement the first months after onset of disease was <0.5 U kg^−1^ day^−1^. However, after one year, her insulin dose increased on MDIs, and at two and a half years of age, she was switched to insulin pump therapy (Table 1). 

The father, now 40 years old, was diagnosed with diabetes at the age of 12 weeks when he was admitted comatose to the hospital with severe DKA. He was rehydrated and treated with insulin and later discharged with an insulin dose of 2 units once daily (unknown weight at onset). Other clinical information is lacking from time of onset. He tested negative for T1D-related autoantibodies checked for the first time after 35 years of disease. At 34 years of age, he was changed from a regimen of two daily insulin injections to MDIs with a following improvement in HbA1c from 9.5% to 7.7% (Table 1). He experiences late diabetic complications of preproliferative retinopathy in a progressive phase bilaterally and with maculopathy unilaterally. At present there was no sign of micro- or macroalbuminuria nor evidence of clinical neuropathy. He is normotensive without medication.

### 3.1. Family Genotyping

**Figure 1:**
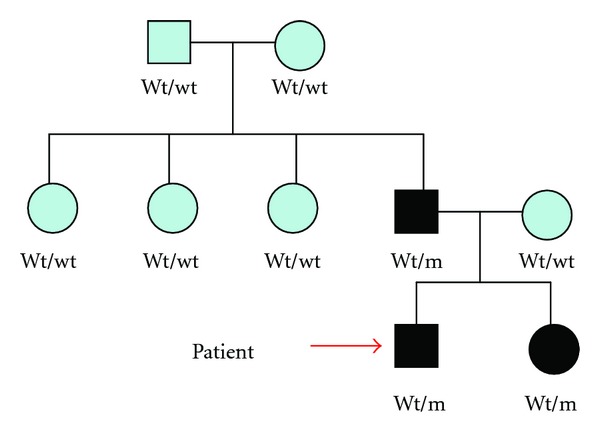
Squares represent male family members, and circles represent female members. Wt = wildtype, Wt/m = wildtype/mutant. Solid squares and circles represent persons with diabetes carrying the C95Y mutation.

The mother of the children, the paternal three aunts, and the paternal grandparents did not carry the mutation in the insulin gene (Figure 1), which presumably originated *de novo* in the father.

## 4. Discussion

**Figure 2:**
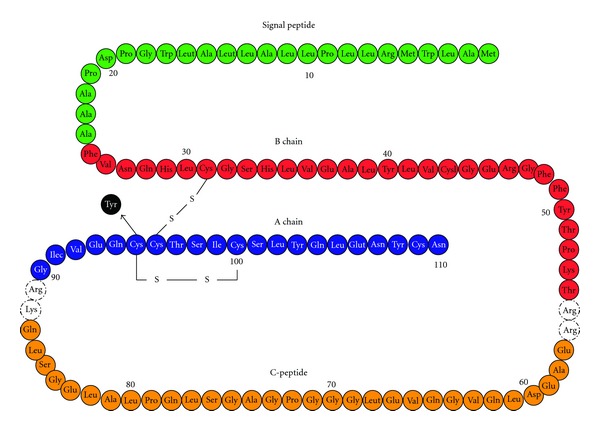
Diagram representing the human preproinsulin molecule marking the location of the C95Y mutation found in the three family members.

We have described the clinical features of a family with three members carrying a heterozygous C95Y mutation of the insulin gene associated with PNDM. Further characterization of the clinical phenotype in the *INS* mutations carriers is useful to distinguish these patients from *KCNJ11* mutations carriers. The C95Y mutation, which is located in the *α*-chain of the preproinsulin molecule, disrupting one of the three invariant disulfide bridges (Figure 2), causing misfolding of the protein. It has previously been suggested that accumulation (and degradation) of abnormally folded insulin in the pancreatic beta cell within the endoplasmic reticulum (ER) causes sustained ER stress and subsequent beta-cell death by apoptosis, resulting in decreased insulin production/secretion that ultimately leads to hyperglycaemia [7, 9–11].

The clinical features in our cases depict a median age at presentation of 119 days; all three cases were delivered at term and had a near-normal birth weight for gestational age, confirming previous studies of *INS* mutation carriers [4, 5, 7, 9, 10]. The heterogeneous presentation in the family, the father showing severe DKA at 12 weeks and the patient presenting with “only” moderate hyperglycemia (serum blood glucose of 9.4 mmol/L) at 28 weeks, suggests a faster pancreatic beta-cell death in the father or a higher pancreatic cell mass of the son. Oslowski and Urano suggested that the unfolded protein response (UPR), functioning as a binary switch between survival and death of the pancreatic beta cells may counteract the signalling pathway to sustained ER stress (leading to apoptosis of the insulin producing cells) [12]. They hypothesized that UPR can, through several mechanisms, restore the disrupted homeostasis within the ER, promoting cell survival. We believe that such a mechanism may be operative in our patient. In addition, parental alertness of diabetes-specific symptoms may have led to early diagnosis with prevention of DKA in the son.

Our three cases needed a low daily insulin dose (less than 0.5 U kg^−1^ day^−1^) until the first six months after diagnosis, but then developed some metabolic instability (Table 1). They all benefited from a more intensive treatment regimen as assessed by their HbA1c value that well compares to our treatment target for HbA1c, which has been set at 7.5% [13]. The median insulin dose requirement in the children is currently 0.50 U kg^−1^ day^−1^ (5 and 3.5 years old), and the father has an insulin need of 0.59 U kg^−1^ day^−1^, which was somewhat lower than the average insulin requirement 0.66 U kg^−1^ day^−1^ for children age 2–9 years reported by the Hvidore Study Group on Childhood Diabetes [13]. 

Interestingly, we detected preproliferative retinopathy in the father, a late diabetic complication that has not been described in patients carrying the *INS *mutation. This result is at variance with one adult case with *INS* gene mutation R89C (also described as R65C) [7], who shows only minor background retinopathy after 40 years of diabetes, and another patient with the same mutation, who shows mild, nonproliferative retinopathy after 32 years of disease (Barbetti F, personal communication). Mortensen et al. found that DKA at diagnosis is related to poorer residual pancreatic beta-cell function 12 months after diagnosis [14]. The father's preproliferative retinopathy might well be due to the combination of severe pancreatic beta-cell destruction at onset, and a limited regeneration of beta cells in the first three decades of his adult life [15] leading to a short remission period and poor glycemic control. However, the father has been dysregulated over a longer period of time on a twice daily insulin regimen, which might have had the major impact on the development of his retinopathy.

In conclusion, screening for a mutation in the *INS* gene must be considered in families with early onset, autoantibody-negative diabetes.

##  Conflict of Interests

The authors declare that they have no conflict of interests.

## Figures and Tables

**Table 1:** Characteristics of the family.

Subject	Patient	Father	Sister
Current age (year)	5 y	40 y	3.5 y
Onset of diabetes			
(i) Age at presentation (week)	28	12	10
(ii) Clinical presentation	Polydipsia/polyuria	Severe DKA*+ comatose	Polydipsia/polyuria
(iii) HbA1c (%)	13.5	N.a.	**5.4
(iv) DKA at presentation	None	Yes	None
(v) Std. HCO3^−1^ (mmol/L)	22	N.a.	24
(vi) Stimulated C-peptide(nmol/l) (reference range 0.40–1.70 nmol/L)	0.82/0.21***	<0.17^§^	0.32
(vii) ^1^GAD, ICA, IA-2, IAA	Negative	Negative^§^	Negative
(viii )Birth weight (gram)	2970	3492	3136
Followup			
Age (years ): HbA1c (%)/insulin dose (U kg^−1^ day^−1^)	1 y: 6.9/0.84 2 y: 8.1/0.61 3 y: 9.5/0.88 4 y: 7.9/0.53 5 y: 7.9/0.52	34 y: 9.5/0.75 35 y: 7.7/N.a. 38 y: 8.0/0.59	1 y: 7.8/0.59 2 y: 7.2/0.69 3 y: 8.6/0.87 3.5 y: 7.7/0.48

^1^Autoantibodies: GAD65 (glutamic acid decarboxylase 65 antibodies), ICA (Islet cell autoantibodies), IA-2A (insulinoma antigen-2). and IAA (insulin autoantibodies). N.a = not available.

*DKA: diabetic ketoacidosis.

**Inaccurate test due to foetal haemoglobin.

***1/13 months after diabetes onset.

^§^measured 35 years after diabetes onset.

## Abbreviations

DCCT: The Diabetes Control and Complications Trial
DKA: Diabetic ketoacidosis
ER: Endoplasmic reticulum
GAD 65: Glutamic acid decarboxylase 65 antibodies
HbA1c: Glycated haemoglobin
IAA: Insulin autoantibodies
IA-2A: Insulinoma antigen-2 antibodies
ICA: Islet cell autoantibodies
K_ATP_: Potassium, adenosine triphosphate
M: Mutation
MDIs: Multiple daily injections
ND: Neonatal diabetes
PNDM: Permanent neonatal diabetes mellitus
TNDM: Transient neonatal diabetes mellitus
T1D: Type 1 diabetes
U kg^−1^day^−1^: Units per kilogram per day
UPR: Unfolded protein response
Wt: Wild type.
